# Tracking dynamic structural changes in catalysis by rapid 2D-XANES microscopy

**DOI:** 10.1107/S1600577521007074

**Published:** 2021-08-12

**Authors:** Saba Alizadehfanaloo, Jan Garrevoet, Martin Seyrich, Vadim Murzin, Johannes Becher, Dmitry E. Doronkin, Thomas L. Sheppard, Jan-Dierk Grunwaldt, Christian G. Schroer, Andreas Schropp

**Affiliations:** a CXNS – Center for X-ray and Nano Science, Deutsches Elektronen-Synchrotron DESY, Notkestraße 85, DE-22607 Hamburg, Germany; b Deutsches Elektronen-Synchrotron DESY, Notkestraße 85, DE-22607 Hamburg, Germany; c Bergische Universität Wuppertal, Gaußstraße 20, DE-42119 Wuppertal, Germany; d Institute for Chemical Technology and Polymer Chemistry, Karlsruhe Institute of Technology, Engesserstraße 20, DE-76131 Karlsruhe, Germany; e Institute of Catalysis Research and Technology, Karlsruhe Institute of Technology, Hermann-von-Helmholtz Platz 1, DE-76344 Eggenstein-Leopoldshafen, Germany; f Department Physik, Universität Hamburg, Luruper Chaussee 149, DE-22761 Hamburg, Germany; g Helmholtz Imaging Platform, Deutsches Elektronen-Synchrotron DESY, Notkestraße 85, DE-22607 Hamburg, Germany

**Keywords:** X-ray microscopy, XANES, QEXAFS, heterogeneous catalysis, *in situ* and *operando*

## Abstract

Many processes and materials in heterogeneous catalysis undergo dynamic structural changes depending on their chemical environment. Monitoring such dynamic changes can be challenging using conventional spectroscopic characterization tools, due to the high time resolution required. Here, a high-resolution 2D X-ray camera operating at 50 Hz full-frame rate was synchronized with a QEXAFS monochromator, enabling rapid spectro-microscopic imaging with chemical contrast over individual pixels. This was used to monitor chemical gradients within a model Pt/Al_2_O_3_ catalyst during catalytic partial oxidation of methane to synthesis gas. The transition from methane combustion (partly oxidized Pt) to combustion-reforming and partial oxidation (fully reduced Pt) was observed by a characteristic reduction front, which progressed from the end of the catalyst bed towards its beginning on the second time scale. The full-field QEXAFS imaging method applied here allows acquisition of entire XANES spectra ‘on the fly’ in a rapid and spatially resolved manner. The combination of high spatial and temporal resolution with spectroscopic data offers new opportunities for observing dynamic processes in catalysts and other functional materials at work. The methodology is flexible and can be applied at beamlines equipped with a QEXAFS or other fast-scanning monochromators and a suitable sample environment for gas phase analytics to allow for catalytic studies at the same time.

## Abstract 2

The local chemical state of a model Pt/Al_2_O_3_ catalyst is visualized *operando* by rapid 2D XANES imaging during the catalytic partial oxidation of methane to synthesis gas.

## 1. Introduction   

Materials used in heterogeneous catalysis can show dynamic structural changes depending on their chemical environment, for example during ignition and extinction of reactions, activation and deactivation, or restructuring through oxidation and reduction processes (Kalz *et al.*, 2017 ▸; Bergmann & Roldan Cuenya, 2019 ▸). As the structure and function of catalysts are closely related, *in situ* and *operando* characterization have gained increasing attention as a key element in understanding heterogeneous catalytic processes (Weck­huysen, 2003 ▸; Topsøe, 2003 ▸; Bañares, 2011 ▸; Chakrabarti *et al.*, 2017 ▸). These methods involve probing the sample (*e.g.* by spectroscopy) under reaction conditions (*in situ*) (Bare *et al.*, 2010 ▸), and ideally while collecting activity data such as conversion, yield or selectivity (*operando*). This allows identification of structure–activity relations, which can in turn help with enhancing the performance of chemical processes, and identifiying catalytic active sites during reactions under realistic conditions (Grunwaldt & Clausen, 2002 ▸; Newton & van Beek, 2010 ▸; Meirer & Weckhuysen, 2018 ▸). In particular, X-ray absorption spectroscopy (XAS) performed at synchrotron radiation sources has proven to be a versatile and powerful *operando* technique in catalysis research, providing structural information on the oxidation state and the local coordination of the active metal sites (Van Bokhoven & Lamberti, 2016 ▸; Doronkin *et al.*, 2017 ▸). At hard X-ray energies, X-ray microscopy is also attracting attention for the ability to perform spatially resolved structural analysis on small catalytic reactors at the millimetre scale (Grunwaldt & Schroer, 2010 ▸; Beale *et al.*, 2010 ▸; Becher *et al.*, 2021 ▸). Further XANES imaging studies requiring even a higher spatial resolution on the nanometre scale were carried out in 2D and 3D both in the soft (van Ravenhorst *et al.*, 2018 ▸) and hard X-ray regime (Meirer *et al.*, 2011 ▸; Gonzalez-Jimenez *et al.*, 2012 ▸). In order to reach a high spatial resolution on the nanometre length scale, this requires the implementation of X-ray optics for either full-field or scanning X-ray microscopy. The latter has the main advantage that besides X-ray absorption other X-ray analytical contrasts such as X-ray fluorescence can be employed (Boesenberg *et al.*, 2018 ▸). In both cases, however, the minimum time to record a full 2D XANES map is often limited to the minute to hour time scale depending on dwell times of the detector or settling times of the scan motors.

Many catalytic processes operate under dynamic conditions such as transient temperatures or gas environments, meaning that a dynamic response of the catalyst can be expected; for example, the light-off curves observed during catalytic emissions control (Casapu *et al.*, 2017 ▸) or partial oxidation of methane. In the latter case, methane is converted in the presence of some oxygen to CO and H_2_. Usually, the reaction ignites over the course of a few seconds, leading to distinctive chemical gradients within the fixed bed of a reactor (Grunwaldt *et al.*, 2006 ▸; Kimmerle *et al.*, 2009 ▸; Stötzel *et al.*, 2012 ▸). Temporally resolved characterization can therefore be important in order to track such changes on a meaningful timescale (Grunwaldt *et al.*, 2001 ▸; Dent, 2002 ▸; Frenkel *et al.*, 2013 ▸). In addition, the structural response of a catalyst system is not necessarily uniform, and therefore spatially resolved characterization of the catalyst under working conditions can also be important (Hannemann *et al.*, 2007 ▸; Korup *et al.*, 2011 ▸, 2013 ▸; Portela *et al.*, 2018 ▸). However, it is generally challenging to perform spatially and time-resolved studies simultaneously. This challenge is increased when including a level of spectroscopic or chemical contrast, and particularly if attempting to measure under *operando* conditions, which requires specific sample environments (Becher *et al.*, 2021 ▸). For example, full-field X-ray microscopy with absorption or phase contrast can effectively probe small catalytic reactors commonly used in catalysis research at the synchrotron. While achieving excellent spatial resolution on the nanometre to micrometre scale, this is typically performed at a fixed energy (Kimmerle *et al.*, 2009 ▸), or in the case of XANES imaging at a relatively small number of distinct energies around an absorption edge of interest (Grunwaldt *et al.*, 2006 ▸; Meirer *et al.*, 2015 ▸; Kalirai *et al.*, 2016 ▸). Here, the number of required energy points depends on the variation in contrast of particular features in the absorption spectrum relative to the noise level in the acquisition. Considering time-resolved spectroscopy, QEXAFS has developed into a key technique, which combines the valuable structural characterization of X-ray absorption spectroscopy (XAS) with rapid data acquisition on millisecond timescales (Frahm, 1989 ▸), although this is typically performed in single point measurements (Grunwaldt *et al.*, 2009 ▸; Frenkel *et al.*, 2013 ▸; Müller *et al.*, 2016 ▸; Nachtegaal *et al.*, 2016 ▸). The combination of these two techniques offers the intriguing possibility to perform rapid spatially resolved spectroscopic imaging over millimetre-scale fields of view, though this has not been demonstrated in practice so far.

In this work, the continuous scanning QEXAFS monochromator of beamline P64 (Bornmann *et al.*, 2019 ▸) at PETRA III (DESY, Hamburg) was synchronized with a high-resolution X-ray camera (PCO.edge 4.2 CLHS) and a quartz capillary microreactor to perform rapid imaging around the Pt *L*_3_ absorption edge of a model Pt/Al_2_O_3_ catalyst at 50 Hz acquisition rate. As the QEXAFS monochromator is based on fast and continuous scanning, and since full-field imaging acquires entire projections in a simultaneous acquisition, this allows for rapid imaging of entire catalytic microreactors and per-pixel recovery of spectroscopic data around the absorption edge of interest. In this case, a single full up or down sweep over the Pt *L*_3_ absorption edge was acquired in 2.8 s, limiting the temporal resolution to the same value in the presented XANES imaging study. Since 10% of all images measured at lowest and highest energy values were not considered, a complete stack of about 80 XANES transmission images was recorded around the absorption edge in a total time of 1.6 s. Catalytic partial oxidation (CPO) of methane to syngas (CO + H_2_) was chosen as a case study. CPO is of interest to the hydrogen economy concept, as it offers a potentially energy efficient route towards generation of syngas as an alternative to large-scale and energy-intensive methane steam reformer facilities (Navarro *et al.*, 2007 ▸; Enger *et al.*, 2008 ▸). In addition, CPO over noble metal catalysts such as Pt, Pd or Rh has demonstrated an interesting oscillatory behaviour, whereby several reaction pathways become active under different temperature conditions, including methane combustion, direct partial oxidation, combustion reforming, among others (Grunwaldt *et al.*, 2006 ▸; Kimmerle *et al.*, 2009 ▸; Stötzel *et al.*, 2012 ▸). This behaviour leads to transient conditions and catalyst structure, which is an ideal proof of principle for rapid spectroscopic imaging.

## 2. Experiment section   

### 2.1. Sample preparation   

The catalyst was prepared by incipient wetness impregnation, using γ-alumina support (Puralox SCFa-230, SASOL, specific surface area approximately 230 m^2^ g^−1^) and hexachloroplatinic acid (H_2_PtCl_6_·*x*H_2_O, Merck) as Pt precursor. The latter was dissolved in water and added dropwise to the support, resulting in a Pt/Al_2_O_3_ catalyst with 2.2 wt% Pt loading according to atomic absorption spectroscopy (Doronkin *et al.*, 2016 ▸). The resulting powder was dried overnight at 70°C and calcined at 500°C for 2 h in static air before further use. The catalyst was pressed and sieved. A fraction with grain sizes between 100 µm and 200 µm was used for the experiment.

### 2.2. 2D XANES imaging   

**Figure 1:**
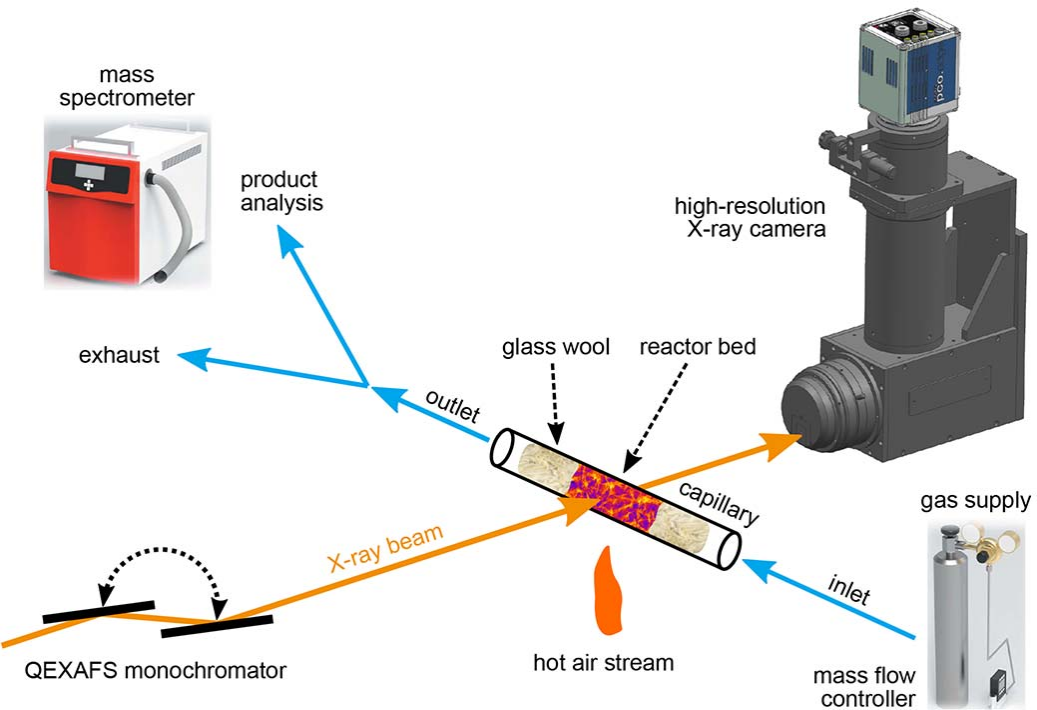
Schematic illustrating *operando* 2D XANES imaging. The catalytic material embedded in a glass capillary is imaged in transmission by a high-resolution X-ray camera at a fast sequence of different energies around a specific X-ray absorption edge.

The XANES imaging experiment was carried out at the advanced spectroscopy beamline P64 at the PETRA III synchrotron radiation source (DESY, Hamburg) (Caliebe *et al.*, 2019 ▸). Since the beamline provides a high monochromatic photon flux and is equipped with a QEXAFS monochromator (Bornmann *et al.*, 2019 ▸), it is perfectly suited for fast XANES experiments. Figure 1 ▸ illustrates the experimental imaging scheme. The catalytic material (2.2 wt% Pt/Al_2_O_3_) was filled as sieved powder into a capillary with an outer diameter of 0.5 mm and fixed in position with glass wool. The length of the catalyst bed was approximately 6 mm. The capillary was installed on a stack of motor stages in order to align it in the horizontal and vertical direction to the X-ray beam. The chemical state of the catalyst was controlled by adjusting the gas flow through the capillary using mass flow controllers (El-Flow, Bronkhorst, The Netherlands), and the temperature using two hot air blowers (LE Mini, Leister AG, Switzerland) positioned below the capillary. The capillary temperature was calibrated in advance using a portable thermometer with a type K thermocouple, to account for differences in temperature from the built-in thermocouple of the gas blowers. In this case for CPO of methane, a gas mixture of 3%CH_4_/1.5%O_2_/He was provided as reactants with a flow rate of 15 mL min^−1^. The reaction products were analyzed by a mass spectrometer (OmniStar GSD 320 O, Pfeiffer Vacuum, Germany) connected to the gas outlet of the catalytic reactor. The setup was checked for leaks to ensure gas tightness before beginning the experiments.

**Figure 2:**
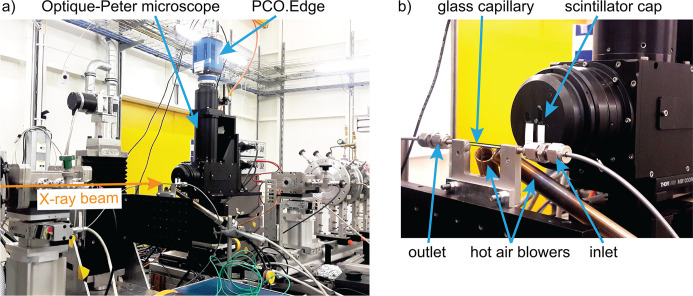
Imaging setup installed at beamline P64. (*a*) Image of the setup showing the Optique-Peter high-resolution X-ray microscope in combination with the PCO.Edge 4.2 CLHS camera. (*b*) The reactor bed embedded in the glass capillary was heated by two hot air blowers. The temperature was measured with thermocouples attached to the nozzle of the air blowers. Reactants flow from the inlet (on the right) towards the outlet (on the left) of the catalytic reactor. The capillary edges are highlighted by black lines for better visibility.

In Fig. 2 ▸ the main parts of the setup installed at P64 are shown, including the quartz capillary reactor, gas supply system, online mass spectrometer, and the high-resolution X-ray camera. The QEXAFS monochromator continuously oscillated in this case around the Pt *L*_3_ absorption edge. In this configuration, sequences of 2D transmission images of the catalytic bed were recorded as a function of energy and time using a high-resolution 2D X-ray camera. The detector is based on a high-resolution X-ray microscope by Optique-Peter imaging the light emitted by an X-ray scintillator screen onto an sCMOS camera (PCO.Edge 4.2 CLHS). As a result of the 4× objective used in combination with a 2.5× ocular, the microscope magnified the imaged area by a factor of 10. With a pixel size of the camera of *p* = 6.5 µm, the effective pixel size in the recorded images was 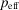 = 0.65 µm. In this configuration the total field of view (FOV) was 1.33 mm × 1.33 mm, well fitting to the X-ray beam size at P64 of about 1 mm in both the horizontal and vertical direction. The sCMOS camera can in principle be operated at a maximum full-frame rate of 100 Hz in rolling shutter mode.

### 2.3. Experimental procedure and data acquisition   

The QEXAFS monochromator was operated independently from the rest of the setup and was continuously cycling over the Pt *L*_3_ absorption edge at a frequency of 0.18 Hz covering an X-ray energy range of about 80 eV in total. The time period to record both a full up and down sweep of the QEXAFS monochromator was therefore 5.6 s, or 2.8 s for just a single up or down sweep over the Pt *L*_3_ absorption edge. Its angular position, which is linked via Bragg’s law to a specific X-ray energy, could be measured by an incremental encoder. This signal was synchronized with the image acquisition of the camera. In this way, for every recorded transmission image an encoder value was obtained at the same time, providing the X-ray energy at which a specific image was taken.

During the experiment the temperature was increased in distinct steps and at each temperature level a sequence of energy-resolved transmission images was recorded. The spatially resolved X-ray absorption spectra were then calculated by taking into account the intensity of the incoming X-ray beam *I*_0_(*E*, *x*, *y*), for which the capillary was moved out of the X-ray beam. These so-called flat-field images were recorded just before the actual measurement with a continuously oscillating monochromator and a total exposure time of 200 s at 50 Hz, *i.e.* about 10000 single frames. In addition, all images were dark-field corrected using a sequence of images obtained with the beam shutter closed in order to account for a constant signal background specific to the camera. In this case, about 500 frames were recorded at 50 Hz, *i.e.* about 10 s total exposure time, and then averaged to create a representative dark-field image to be subtracted from the imaging data.

**Figure 3:**
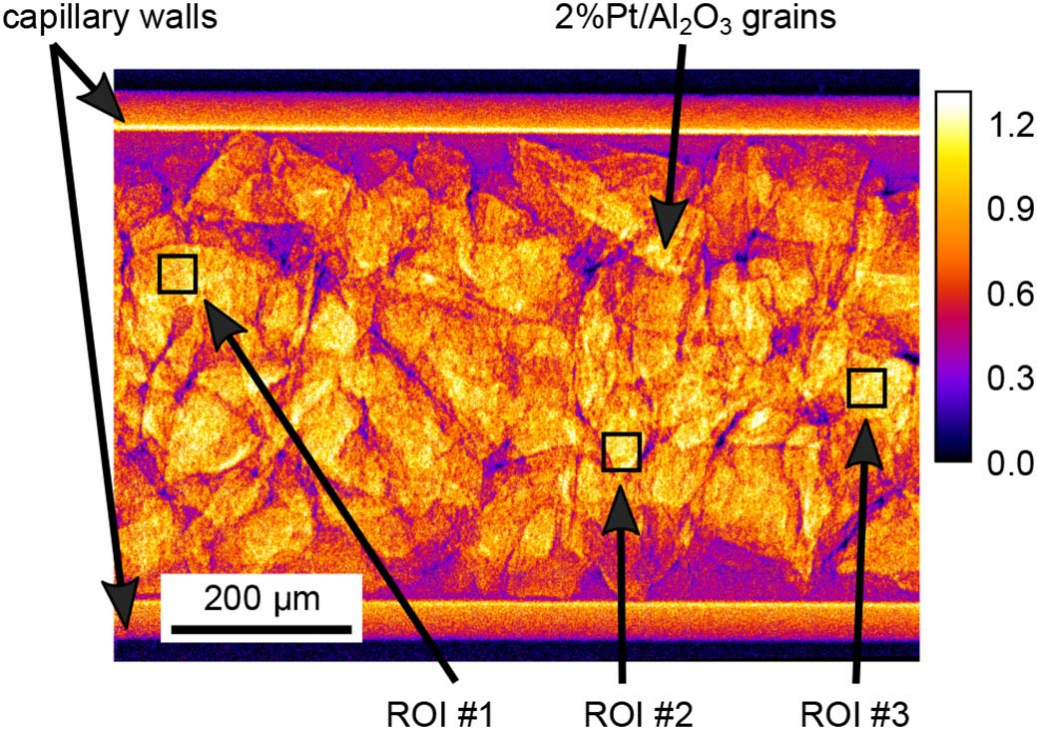
Dark- and flat-field-corrected 2D absorption image of the glass capillary containing the catalyst material measured at an X-ray energy of *E* = 11.54 keV. The catalyst consisted of 2.2 wt% Pt/Al_2_O_3_-grains.

The transmission images of the catalytic reactor bed were further processed using the Lambert–Beer law describing X-ray absorption in matter, *i.e.*

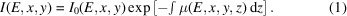
The projected attenuation of the X-rays in the beam direction 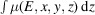 can be retrieved as a function of energy *E* and lateral position (*x*, *y*) inside the catalyst bed,
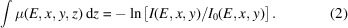
In Fig. 3 ▸ an exemplary dark- and flat-field-corrected absorption image of the catalytic reactor bed is shown, which was obtained by the aforementioned data processing.

**Figure 4:**
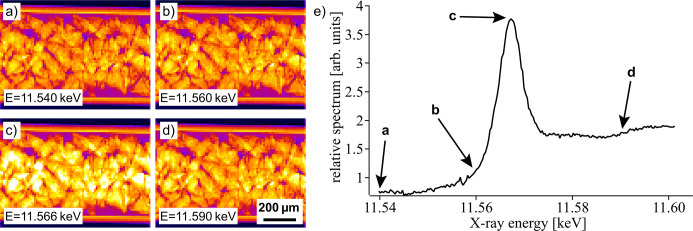
(*a*)–(*d*) Binned absorption images of the catalytic bed measured at different X-ray energies and at a temperature of *T* = 127°C. (*e*) Corresponding XANES spectrum obtained by averaging over the three ROIs indicated in Fig. 3 ▸.

However, due to the relatively high noise level in single pixels, the images had to be binned by a factor of 16 × 16 in order to enhance the local XANES signal such that linear fitting routines could be applied. In Figs. 4 ▸(*a*)–4(*d*) a sequence of such binned images measured at selected energies is shown. They highlight that maximum absorption occurs at the Pt *L*_3_ absorption edge at 11.566 keV.

The XANES spectrum in Fig. 4 ▸(*e*) was generated by averaging the signal in the three ROIs shown in Fig. 3 ▸ for all images recorded during a single oscillation of the QEXAFS monochromator. At this early time in the experiment the Pt in the reactor was still fully oxidized as supported by the strong white line of the XANES curve.

### 2.4. Data sorting and further processing   

Since the QEXAFS monochromator and X-ray camera were operated independently, the measured images had to be re-organized after data acquisition. This is especially important for the determination of an appropriate flat-field image as the incoming 2D intensity distribution of the X-ray beam changes as a function of X-ray energy. The flat-field images were therefore sorted in 200 energy bins, each with a width of about 0.5 eV. By averaging all images of a single bin a representative flat-field image corresponding to a specific X-ray energy was obtained. Typically, a bin contained more than 10 single flat-field images.

**Figure 5:**
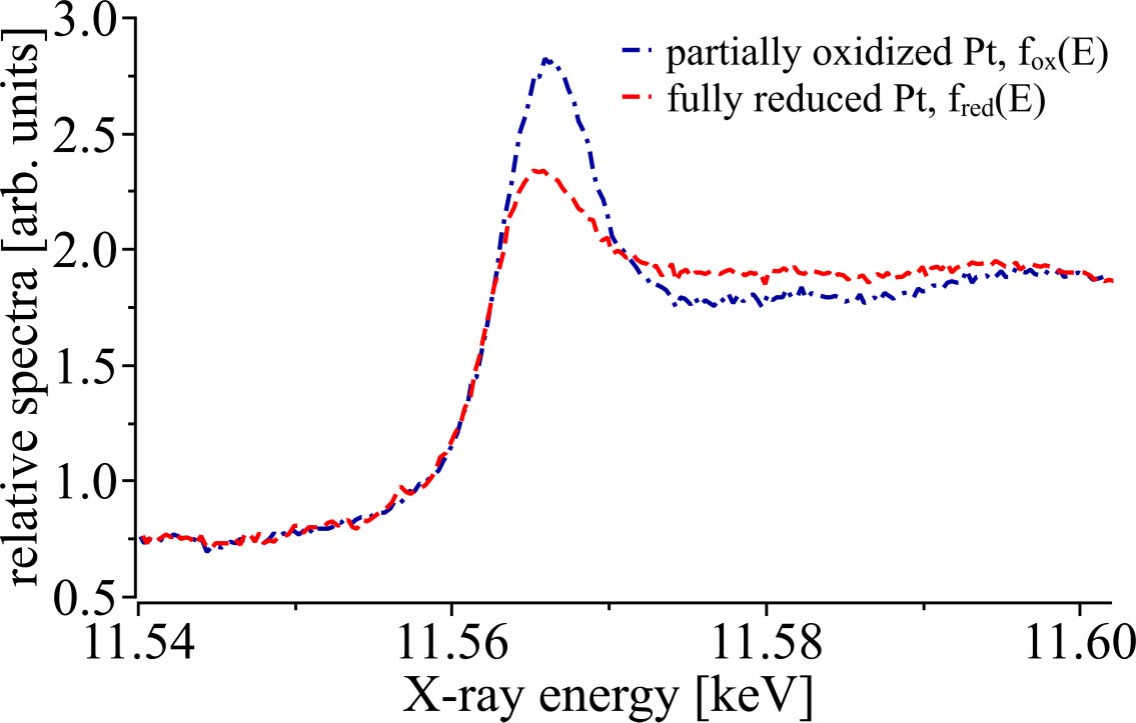
Reference spectra extracted from sequences of X-ray transmission images of the partially oxidized (dash-dotted blue line) and fully reduced (dashed red line) catalytic reactor bed.

Reference spectra representing the partially oxidized Pt and the fully reduced state of metallic Pt were obtained from scans measured at the very beginning of the ignition phase and the very end of an experimental run at temperatures of *ca.* 290°C and 370°C, respectively. These reference spectra were extracted and averaged from three different regions of interest (ROIs) of the sequence of XANES transmission images as indicated in Fig. 3 ▸. The corresponding XANES reference curves are shown in Fig. 5 ▸. It is this level of chemical contrast that enables us to visualize the local chemical state of Pt within the reactor bed during the ignition of the chemical reaction.

In XANES spectroscopy different techniques for data analysis are well established and used depending on data quality, the extent of prior knowledge on the studied system such as the availability of reference spectra and the information to be extracted. For systems with a low understanding of the underlying chemical processes and without clearly defined reference states, principal component analysis is well suited. Often, there is a prior guess about the number and nature of species but obtaining reference spectra comparable with the studied data is not possible or not practical. In this case, internal reference spectra and their weights can be obtained by chemometric tools such as multivariate curve resolution – alternating least-squares (MCR-ALS) (de Juan *et al.*, 2014 ▸) and non-negative matrix factorization (NNMF) (Borodina *et al.*, 2015 ▸). The obtained weights can be related to the concentration of individual species if purity of the internal references can be proven, *e.g.* by comparing spectral features with the literature data. Less commonly used machine learning tools are required if the low data quality makes comparison with reference data not possible or if intermediate states could not be treated as a linear combination of spectra of reference states, but there is good understanding of the system, which allows producing theoretical datasets for training neural networks. The most common cases for using machine learning are XANES datasets in which no chemical transformation occurs but only the nanoparticle size is changing (Timoshenko *et al.*, 2016 ▸) or to analyze EXAFS datasets (Qian *et al.*, 2021 ▸).

As fits with the aforementioned reference spectra can systematically explain the observed spectra within the uncertainty given by the noise, linear combination analysis (LCA) was chosen to fit the measured XANES spectra using the aforementioned reference curves for the partially oxidized and reduced state of Pt, 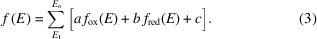
The amount of oxidized Pt, reduced Pt and constant background (absorption by other elements) was then retrieved by a least-squares search yielding the fit factors *a*, *b* and *c* in equation (3) for every pixel of the 2D image. The LCA data analysis code is written in Python using the minimize-function of the *SciPy* package (SLSQP method), which can provide in principle quantitative information. Since the XANES imaging data were acquired continuously in order to make optimal use of the available photon flux, a total of more than 500000 single transmission images were recorded yielding about 3800 XANES image sequences and, therefore, about 20 million single-pixel XANES spectra were fitted by LCA in this study.

In this way, information on the local chemical state was obtained for every XANES image sequence measured in about 2.8 s total exposure time and allowed us to follow the dynamics of the chemical reaction inside the catalyst bed as a function of time and applied temperature.

## 3. Results   

The reaction of methane and oxygen can proceed via different chemical paths, in this case depending on the temperature of the system. In order of increasing temperature firstly a small amount of methane combustion can be expected. This can be followed by a two-step catalytic combustion-reforming (CCR) process, subsequently forming CO and H_2_ (Bharadwaj & Schmidt, 1995 ▸; Schwiedernoch *et al.*, 2003 ▸). Another alternative pathway to syngas formation is the direct partial oxidation (DPO) of methane (York *et al.*, 2003 ▸). The transition between these paths is known to affect the oxidation state of Pt, whereby combustion may occur on an oxidized surface, and reforming in the absence of O_2_ leads to a reduced state. Monitoring the oxidation state can therefore provide an indication of the dominant reaction path (Kimmerle *et al.*, 2009 ▸), here denoted by ‘*A*’ and ‘*B*’, respectively:

(*A*) *Catalytic combustion and reforming (CCR)*

(1) Combustion of methane:
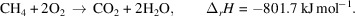



(2) Reforming of methane: 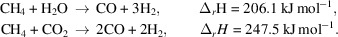



(*B*) *Direct partial oxidation (DPO)*

(1) Partial oxidation of methane:
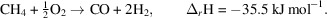
During this measurement the reactor bed was heated up slowly (step by step) and several reaction zones with distinct changes in oxidation state were observed: (i) pre-ignition (from 50°C to 290°C) – the oxidation state of Pt slowly changes from the fully oxidized to the partially oxidized state homogeneously over the catalytic bed; (ii) CPO ignition point (around 290°C) – during the start of the chemical reaction towards CO and H_2_ a sharp interface appears between reduced and oxidized Pt moving from the outlet towards the inlet; (iii) several temperature steps above the ignition point (from 290°C to 370°C) – continuous reduction of Pt and increase in CO and H_2_ selectivity.

The reactor was heated up slowly from room temperature to the ignition temperature at around 290°C step by step. At each temperature step, the catalyst was allowed to thermally stabilize before starting the image data acquisition.

**Figure 6:**
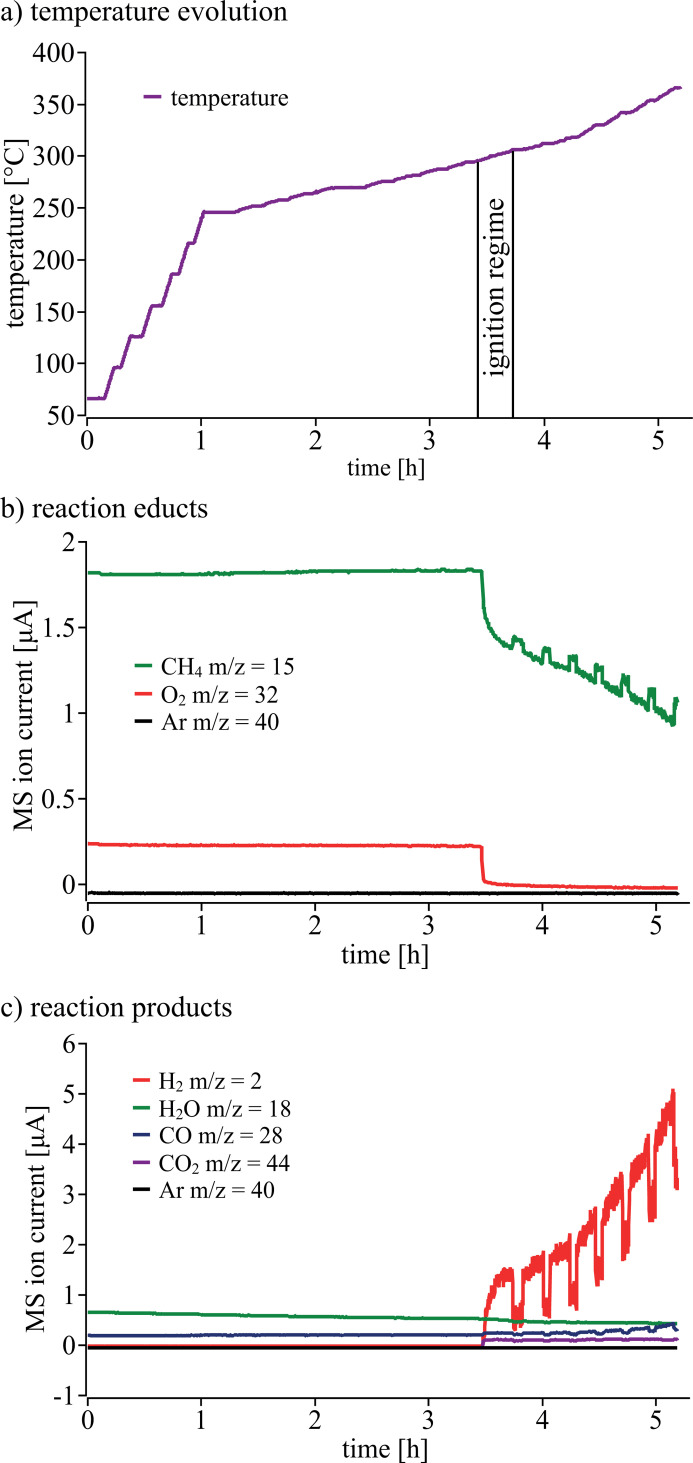
(*a*) Temperature evolution of the catalytic bed. (*b*, *c*) Reaction educts and products measured by mass spectrometry, respectively. Note that the periodic steps in the gas composition stem from moving the microreactor out of the X-ray beam (see text).

In Fig. 6 ▸(*a*) the temperature evolution during the experiment is shown. At the beginning the temperature was fast increased in bigger steps of 25°C up to about *T* = 250°C. In order not to miss the ignition of the chemical reaction the step size was reduced to 10°C afterwards. At each temperature level a sequence of absorption images was collected yielding full 2D XANES spectra. The ignition then started about 3.5 h after the beginning of the experiment at about 290°C, which was associated with a sudden consumption of CH_4_ and O_2_ and production of H_2_, CO and CO_2_ as shown by mass spectrometry [see Figs. 6 ▸(*b*) and 6 ▸(*c*)]. The seven distinct jumps in conversion are related to moments in which the capillary was slightly moved out of the X-ray beam and away from the gas blowers in order to collect flat-field images, leading to a temperature drop and a lower conversion. Prior to ignition, no significant conversion of methane was observed. This is in agreement with previous studies where Pt alone shows relatively low activity for methane combustion (Becker *et al.*, 2007 ▸).

**Figure 7:**
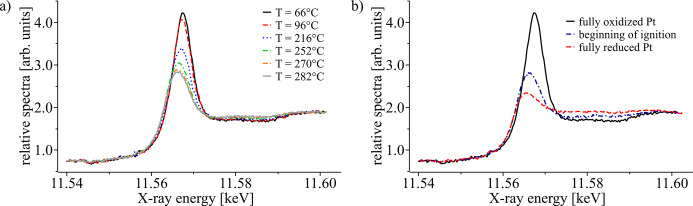
(*a*) XANES spectra measured at different temperatures during the pre-ignition phase indicating a gradual reduction of Pt over time. (*b*) Comparison of XANES spectra measured at the very beginning, during the ignition of the chemical reaction and the very end of the experiment.

In Fig. 7 ▸(*a*) XANES spectra during the pre-ignition phase are shown. The spectra were obtained by averaging over the three ROIs indicated in Fig. 3 ▸. XANES spectra measured at the beginning of the experiment are characterized by an intense absorption peak (white line), indicating that the Pt is fully oxidized and in the Pt^4+^ state. While increasing the temperature from *T* = 66°C to *T* = 282°C, the strength of the white line gradually decreases indicating a slow homogeneous reduction of Pt particles before the actual start of the ignition. This is in agreement with relatively low activity towards methane combustion over Pt/Al_2_O_3_ catalysts, since at the same time no appreciable methane consumption was observed by mass spectrometry. Methane combustion on Pt is often associated with an oxidized surface state. Prior to ignition, heating has been shown to gradually remove oxygen species from the surface of Pt, leading to a gradual decrease in the white line observed. This is followed by ignition, and the complete reduction of Pt, shown by XAS (Becker *et al.*, 2007 ▸; Kimmerle *et al.*, 2009 ▸) and infrared spectroscopy (Chen *et al.*, 2018 ▸).

In Fig. 7 ▸(*b*) a comparison of XANES profiles obtained at low temperature (black line, fully oxidized Pt), around the ignition point (blue dashed-dotted line, partially oxidized Pt), and at the very end of the experiment (red dashed line, fully reduced Pt) is shown. In order to visualize the fast chemical reduction of Pt at the very beginning of the ignition, we used the partially oxidized and fully reduced spectra as reference curves for data fitting using LCA (see Section 2.4). In this way, information on the local Pt oxidation state in the catalyst bed can be derived and displayed as a function of time.

**Figure 8:**
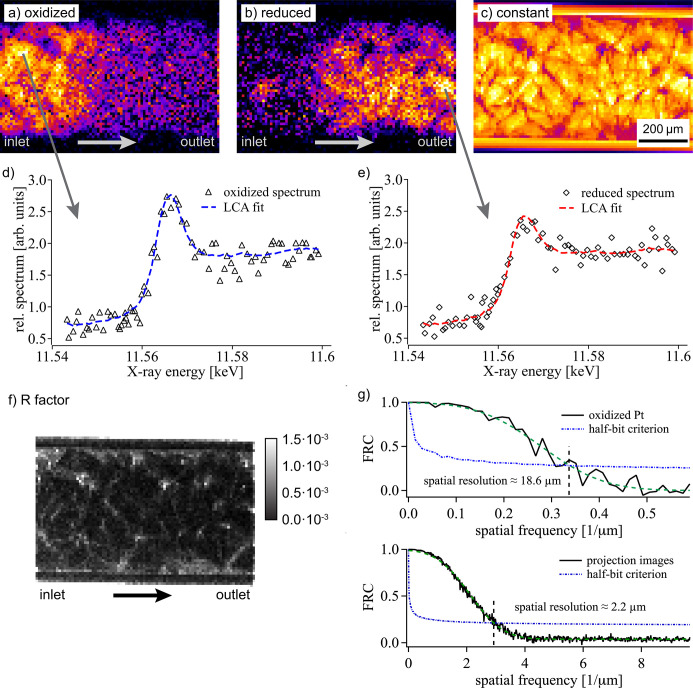
(*a*) Amount of oxidized Pt, (*b*) reduced Pt and (*c*) the distribution of other materials measured at a temperature of approximately 290°C. (*d*, *e*) Single XANES profiles extracted from areas as indicated in the upper images. (*f*) *R*-factor map indicating differences in fit quality depending on the amount of Pt in a pixel (scaled between 0 and 1.5 × 10^−3^ to enhance visibility). (*g*) Fourier-ring-correlation analysis using two subsequent XANES images before ignition yields a spatial resolution of 18.6 µm. In comparison, the spatial resolution of the raw transmission images is about 2.2 µm as indicated in the lower FRC spectrum.

In Fig. 8 ▸ an exemplary result of LCA data fitting is summarized, showing images of the fitting coefficients *a*, *b* and *c*, corresponding to the amount of oxidized Pt [see Fig. 8 ▸(*a*)], reduced Pt [see Fig. 8 ▸(*b*)] as well as a constant offset not related to Pt [see Fig. 8 ▸(*c*)]. The glass capillary, which obviously does not contain any Pt, is therefore only visible in Fig. 8 ▸(*c*). Exemplary plots of single-pixel XANES profiles are shown in Figs. 8 ▸(*d*) and 8 ▸(*e*) referring to rather oxidized and reduced areas of the catalytic reactor, respectively. Here, a distinct interface between oxidized and reduced Pt could be observed within the catalytic bed.

The quality of data fitting by LCA analysis depends on the signal-to-noise ratio in each pixel as illustrated in Fig. 8 ▸(*f*). It shows an image of the typically used *R*-factor defined by
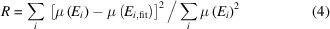
(Meirer *et al.*, 2011 ▸), where μ(*E*_*i*_) and μ(*E*_*i*,fit_) are the measured and fitted values of the absorption coefficient at the measured X-ray energy values *E*_*i*_, respectively. In this case, the *R*-factor varies between about 0.5 × 10^−3^ and 3 × 10^−3^ with highest values in areas with low imaging contrast, *i.e.* less Pt content. The spatial resolution in the presented images were evaluated by Fourier ring correlation (FRC) (van Heel & Schatz, 2005 ▸; Banterle *et al.*, 2013 ▸) using two subsequent images of the time series. It shows a decrease in spatial resolution from 2.2 µm (half-period) for the raw imaging data [see Fig. 3 ▸ and lower FRC plot in Fig. 8 ▸(*g*)] to about 10 µm for the constant background image [see Fig. 8 ▸(*c*)], and slightly better than 20 µm in images of the oxidized or reduced Pt distribution [see upper FRC plot in Fig. 8 ▸(*g*)]. The contribution of Pt to the total absorption is about an order of magnitude lower as compared with the bulk material, leading to a reduced imaging contrast.

**Figure 9:**
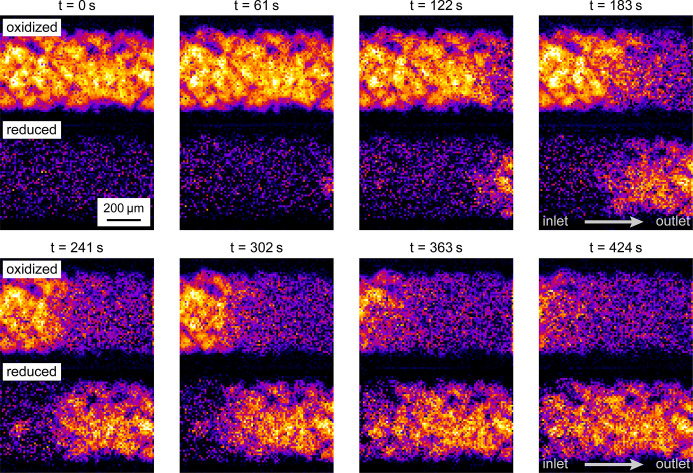
2D distribution of oxidized Pt (upper images) and reduced Pt (lower images) during the ignition phase. The reduction of Pt starts at the outlet and then moves fast towards the inlet of the catalytic reactor.

The XANES imaging data can then be arranged in a sequence in order to visualize the local oxidation state of the reactor as a function of time. In Fig. 9 ▸ a summary of these results is shown. It indicates that during the ignition of the chemical reaction the reduction of the material starts at the outlet of the catalytic reactor and the relatively sharp interface between oxidized and reduced Pt then moves rapidly towards the inlet. It passes through the field of view of about 1 mm in about 7 min. In this way we can visualize the oxidation state of the chemical reactor as a function of position and time providing a detailed insight into the kinetic changes occurring in such processes.

Although the sample was illuminated continuously with a high photon flux, issues related to radiation damage were not observed and the sample stability was uncritical at this length scale.

The key advance here is the ability to measure 2D images of a large section of the catalyst bed, with high time resolution, and additionally with energy-resolved XANES spectra. While previous X-ray spectro-microscopy studies have focused on rapid imaging at a single energy, or relatively slow imaging at multiple energies across the XANES region, here all of the above are combined. The ability to perform rapid spatially and energy-resolved measurements allows to more accurately follow the reaction progress via spectroscopic imaging. Demonstrated here with a straightforward case study showing the gradient between oxidative and reductive processes, this concept may now be exploited to investigate other complex chemical systems. In particular, rapid spectroscopic analysis may be valuable when coupled with kinetic investigations under fluctuating conditions of temperature or reaction mixture. This may help to deconvolve specific processes or reaction steps from a complex series of events. The time scale demonstrated here makes it feasible to probe numerous conditions during a single measurement campaign at a synchrotron radiation source, potentially allowing a high-throughput analysis of chemical environments and reaction conditions for individual samples. Crucially, the methodology shown here is flexible and can easily be applied at other XAS or QEXAFS beamlines.

## 4. Conclusions   

We have demonstrated that rapid 2D-XANES imaging can be implemented at beamline P64 at PETRA III. By using the fast QEXAFS monochromator in combination with a high-resolution X-ray camera, full XANES movies were recorded with a time resolution of 2.8 s and a local sensitivity on the 10 µm scale, yielding temporally and spatially resolved chemical information of the catalytic reactor. In principle, current QEXAFS technology allows for quicker energy scanning on the 100 ms time scale and even below and, therefore, if signal levels are sufficiently high and faster 2D X-ray cameras become available, the time resolution can be further improved. Here, we examined the chemical state within a Pt/Al_2_O_3_ catalyst during catalytic partial oxidation of methane to synthesis gas. During the ignition of the chemical reaction a distinct gradient between partially and fully reduced Pt appeared, which moved fast from the end to the beginning of the catalyst bed on a second timescale. To date, fast 2D X-ray imaging is rather seldom applied in this scientific field, but offers new possibilities for observing dynamic processes in catalysts or other functional materials at work (*in situ* and *operando*). Due to the relatively low photon sensitivity of the X-ray detector the images had to be binned by a factor of 16 limiting the spatial resolution to slightly better than 20 µm in this case. However, future detector upgrades using, for example, pixel detectors will considerably improve the sensitivity pushing the temporal and spatial resolution in such *operando* catalysis experiments towards the millisecond timescale and the micrometre range, respectively.

## Supplementary Material

Single XANES image sequence. DOI: 10.1107/S1600577521007074/ye5006sup1.mov

Reaction kinetics in combination with XANES imaging results (full experimental sequence). DOI: 10.1107/S1600577521007074/ye5006sup2.mov

Reaction kinetics in combination with XANES imaging results during ignition (enhanced contrast). DOI: 10.1107/S1600577521007074/ye5006sup3.mov
